# CFD investigations of a shape-memory polymer foam-based endovascular embolization device for the treatment of intracranial aneurysms

**DOI:** 10.21203/rs.3.rs-5014601/v1

**Published:** 2024-10-18

**Authors:** Tanner Cabaniss, Ryan Bodlak, Yingtao Liu, Geoffrey Colby, Hyowon Lee, Bradley Bohnstedt, Rinaldo Garziera, Gerhard Holzapfel, Chung-Hao Lee

**Affiliations:** The University of Oklahoma Norman; The University of Oklahoma Norman; The University of Oklahoma Norman; UCLA Health; Purdue University West Lafayette; Indiana University School of Medicine Indianapolis; University of Parma; Graz University of Technology; University of California Riverside

**Keywords:** Computational fluid dynamics, intracranial aneurysms, cerebral aneurysms, shape memory polymers, patient-specific therapeutics

## Abstract

The hemodynamic and convective heat transfer effects of a patient-specific endovascular therapeutic agent based on shape memory polymer foam (SMPf) are evaluated using computational fluid dynamics studies for six patient-specific aneurysm geometries. The SMPf device is modeled as a continuous porous medium with full expansion for the flow studies and with various degrees of expansion for the heat transfer studies. The flow simulation parameters were qualitatively validated based on the existing literature. Further, a mesh independence study was conducted to verify an optimal cell size and reduce the computational costs. For convective heat transfer, a worst-case scenario is evaluated where the minimum volumetric flow rate is applied alongside the zero-flux boundary conditions. In the flow simulations, we found a reduction of the average intra-aneurysmal flow of > 85% and a reduction of the maximum intra-aneurysmal flow of > 45% for all presented geometries. These findings were compared with the literature on numerical simulations of hemodynamic and heat transfer of SMPf devices. The results obtained from this study can serve as a guide for optimizing the design and development of patient-specific SMPf devices aimed at personalized endovascular embolization of intracranial aneurysms.

## 1. Introduction

Intracranial aneurysms (ICAs), which affect approximately 4% of the American population (Keedy et al. 2006), are saccular protrusions that arise form focal dilation of the arterial wall (Toth et al. 2018). It has been found that 85% of ICAs develop within *the circle of Willis*, the complex central cranial vasculature (Keedy et al. 2006). Although there are different classifications of ICAs, namely saccular, fusiform, dissecting, and mycotic, this study focuses exclusively on saccular aneurysms as they account for approximately 90% of ICAs (Keedy 2006). The primary risk of ICAs is the possibility of rupture, which can further lead to subarachnoid hemorrhage: a bleeding in the intracranial cavity that leads to a variety of life-threatening conditions such as severe brain damage, long-term morbidity and/or mortality (Schievink et al. 1997). While the causative physiological mechanisms for ICA rupture are not yet fully understood, current literature presents correlations with multiple factors, including age, hypertension, smoking history, alcohol abuse, hypercholesterolemia, and carotid stenosis (Wagner et al. 2005).

The success rate of ICA treatment is mainly measured using the Raymond-Roy Occlusion Classification scale. This classification evaluates the degree of occlusion of the aneurysm sac into three distinct classes: Class I – complete obliteration; Class II – residual neck; and Class III – residual aneurysm (Roy et al. 2001). Complete obliteration, also called complete occlusion, is defined as the cessation of intra-aneurysmal blood circulation, with the majority of blood flowing through the parent artery as if the aneurysm were not present.

Guglielmi detachable coils (GDCs), developed in the 1990s (Guglielmi et al. 1992), have long been considered the gold standard for *endovascular* treatment of ICAs. According to a retrospective study by Gonda et al. (2014) over 2,509 unruptured aneurysms, while GDCs have a 30-day postoperative mortality rate lower than that of highly invasive microsurgical clipping (1.1% vs. 2.3%), in the long term they have unsatisfactorily lower rates of complete occlusion (i.e., Raymond-Roy Occlusion Classification Class I). Another retrospective study by Murayama et al. (2003) reported that traditional coiling had an astonishing complete closure rate of 55%, compared to complete occlusion rates of > 90% with microsurgical clipping (Natarajan et al. 2008; Schwartz et al. 2018; Choi et al. 2020).

To address this critical shortcoming in aneurysm occlusion, new endovascular devices have been developed over the past 2–3 decades. These devices consist of direct improvements to GDCs through the introduction of hydrogel and shape memory polymer (SMP)-based coatings as well as novel endovascular devices such as stents and flow diverters (Alderazi et al. 2014; Taschner et al. 2018; Herting et al. 2019). While stent-based devices have demonstrated improved complete occlusion rates of up to 83.3% (Hanel et al. 2023), flow diversion via stents is only useful in the treatment of sidewall aneurysms, so bifurcation aneurysms continue to be treated primarily via coil-based techniques. Although these results provide significant room for improvement, new endovascular technology may be the answer to achieving comparable rates of complete occlusion to microsurgical clipping procedures without the associated procedural risks of a craniotomy.

This emerging technology is based on the use of shape memory polymer foams (SMPf) – a subgroup of SMP materials that possess a high degree of porosity while maintaining the shape recovery properties of typical shape memory polymers (Pineda-Castillo et al. 2022). These properties allow the SMPf to be compressed into a microcatheter, implanted into an aneurysm cavity, and subsequently expand and recover to its original programmed geometry via shape recovery activation, thereby occluding the aneurysm sac. The SMPf have been fabricated with both closed and open cell architectures, the latter enabling higher compression ratios required for endovascular techniques (Wang et al. 2019). Moreover, recent work has used discrete lattice architectures to enable further degrees of structural optimization (Pineda-Castillo et al. 2023). To trigger the shape recovery of SMPf through Joule heating, conductive polymer coatings and nanomaterials such as carbon nanotubes were introduced to increase the electrical and thermal conductivity of the fabricated SMPf (Pineda-Castillo et al. 2020; Pineda-Castillo et al. 2021). Horn et al. (2017) conducted *in vivo* studies on porcine side-wall aneurysms that demonstrated the promise and efficacy of this therapeutic technique. However, several inquiries remain regarding the optimal geometric considerations of these foams. These salient challenges revolve around crucial aspects of foam design such as mechanical stability, optimal configurations to reduce intra-aneurysmal residual circulation, and promotion of platelet aggregation to improve coagulation processes.

One of the earliest applications of computational fluid dynamics (CFD) in aneurysm research came from Gonzalez et al. (1992), who used pulsatile flow and a non-Newtonian fluid model to simulate blood flow through an aneurysm in the middle cerebral artery. By 2004, only eleven additional articles had been published on the topic, but research efforts have increased rapidly since then, as a study by Saqr et al. (2020) found 795 publications on ICA hemodynamics using CFD as the primary *in silico* modeling tool. While there is a wealth of information about hemodynamics in ICAs and the effect of other devices (e.g., stents, coils, etc.), only limited efforts have been made to investigate the impact of SMPf-based devices for *personalized* treatment of ICAs.

The aim of this study is therefore to use CFD to examine the hemodynamic influence of SMPf implantation in “patient-specific” aneurysm geometries. Specifically, our aim is to quantify the reduction in intra-aneurysmal blood flow caused by the SMPf device and to evaluate the convective heat transfer to the surrounding arterial tissues during initiation of shape recovery as well as successive device expansion. The results of this work could also lead us to a deeper understanding of the relevant intricacies of SMPf devices for patient-specific endovascular ICA therapeutics.

The remainder of this paper is organized as follows: in Section 2, we describe the development of a CFD-based approach to analyze the hemodynamics of untreated ICAs and aneurysms implanted with an SMPf-based device, as well as the heat transfer mechanisms associated with activation of shape recovery. In Section 3, we present the results of the models and validate their efficacy using an example from the established literature. In Section 4, we will further assess the advantages and limitations of the patient-specific SMPf device when compared to other established endovascular techniques, followed by some concluding remarks.

## 2. Methods

### 2.1 Geometry Preparation

**Figure 1:**
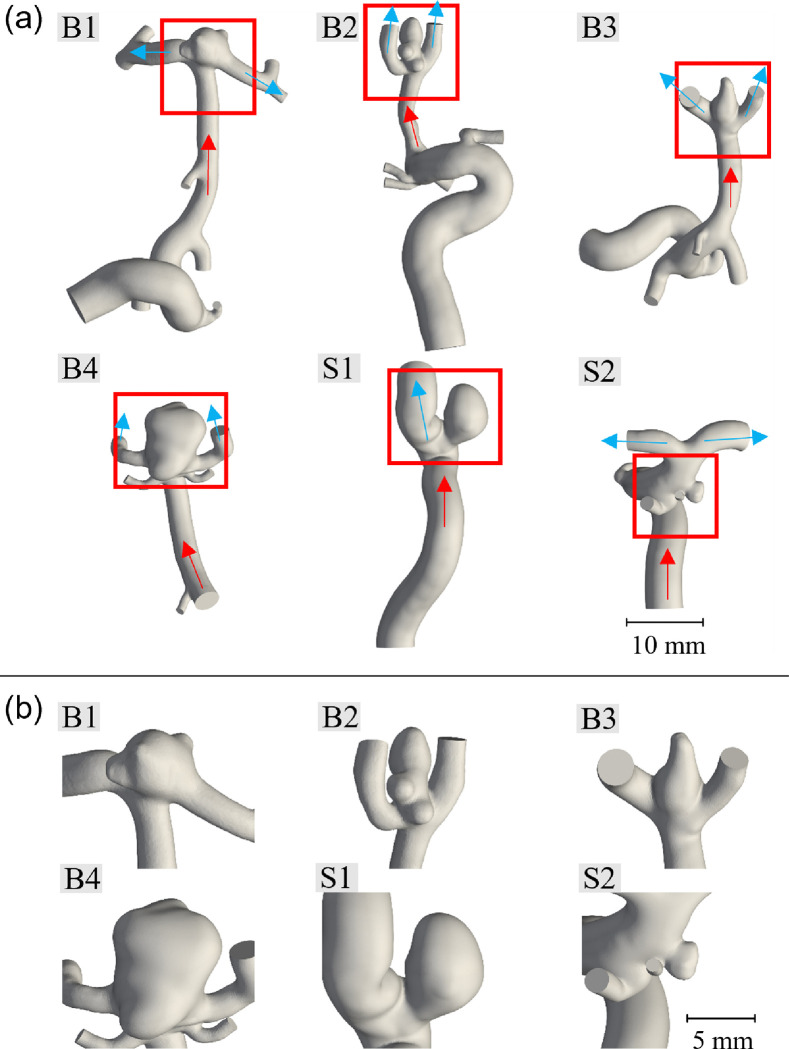
Patient-specific vasculature and aneurysms: (a) segmented vasculature for each geometry with inflow (red arrows) and outflow (blue arrows); (b) magnified view of the patient-specific aneurysms (sizes are to scale relative to each other; B = bifurcation aneurysm; S = side-wall aneurysm).

Eleven anonymized, patient-specific 3D rotational angiography datasets were acquired from the University of Oklahoma Health Sciences Center in 2018 and 2019 (IRB #7932). Of these datasets, nine had sufficient resolution and were selected for image segmentation. These were successively narrowed down to five unique geometries from the collected data; three bifurcation and two sidewall aneurysms as well as another geometry from the literature (Valen-Sendstad et al. 2018) for our numerical modeling/simulation. The four segmented geometries excluded from this study were structurally similar to the geometries presented. Image segmentation was performed in Amira (Thermo Fisher Scientific, Waltham, MA) with 0.108 × 0.108 × 0.5 mm rectangular prism voxels. The segmented patient-specific ICA lumen surface was then imported into Meshmixer (Autodesk, San Rafael, CA) as an STL file and slightly smoothed to remove localized peaks and kinks due to noise in the image data. The vessels were then cut normal to their cross sections to form inlets and outlets, while the outlets were maintained at approximately twice the vessel diameter once their respective directions were established, and the inlets were cut at least seven vessel diameters from the aneurysm sac, as suggested by the literature convention (Moyle et al. 2006; Hua et al. 2015). These cuts were made to reduce the computational costs while maintaining validity of the flow characteristics throughout the studied system (Hodis et al. 2015). The full extent of the modeled vasculature for each geometry and the respective more detailed views of the ICAs are shown in Fig. 1, while the main geometric characteristics of the modeled aneurysms are summarized in Table 1. Here, the bifurcation aneurysm B1 was taken from the 2015 International Aneurysm CFD Challenge (Valen-Sendstad et al. 2018).

### 2.2 Simulation Parameters

Since one of the goals of this study is to support the development of individually-optimized SMPf devices (Kunkel et al. 2018;Wang et al. 2019), steady-state simulations were performed in OpenFOAM 8 (Weller et al. 1998), assuming that the greatest degree of blood flow into the SMPf device occurs during systole. Moreover, because steady-state simulations with systolic flow tend to overestimate the velocity field (Geers et al. 2014), this consideration provided an upper bound on the worst-case flow that the SMPf device and the treated aneurysm would experience. Accordingly, steady-state solutions were achieved using the SIMPLEC (Semi-Implicit Method for Pressure Linked Equations-Consistent) algorithm and intra-aneurysmal blood flow residuals were monitored to ensure convergence. The meshes were constructed predominantly from hexahedron cells, while prismatic and tetrahedral cells were incorporated along the boundaries with a growth factor of 1.2 in the 12–20 inflation layers, resulting in mesh sizes from 4.4 × 10^6^ to 7.9 × 10^6^ cells. The results of the mesh selection process and convergence study are presented in Sections 2.6 and 3.1.

In these simulations, the blood flow was modeled to be an incompressible Newtonian fluid with a dynamic viscosity *μ* = 3.65 × 10^−3^ Pa·s, a density *ρ* = 1050 kg/m^3^ and a kinematic viscosity *υ* = 3.5 × 10^−6^ m^2^/s (Hasgall et al. 2018). No material was assigned to the vessel walls, but they were treated as rigid according to convention, with a no-slip boundary condition applied along the walls (Berg et al. 2019).

Next, the inlet boundary conditions were defined using the peak systolic volumetric flow rate (VFR) values estimated from population-averaged flow waveforms for the ICA and basilar artery reported in the literature for a normal, young cohort: 9.26 mL/s and 5.63 mL/s, respectively (Horn et al. 2017). Generalized waveforms for older adults were avoided due to the age range of the present cohort was unknown, although both datasets would likely have produced similar flow patterns and wall shear stresses (WSS) (Xu et al. 2018). According to Chnafa et al. (2018), using the splitting method outlined above, VFRs were prescribed on all but one outlet, typically the smallest. The unassigned outlet was left in the zero pressure condition while a zero-gradient pressure boundary was applied to all other outlets.

Finally, there is increasing evidence that transitional or turbulent flow occurs in physiological blood flow (Saqr et al. 2020) and that turbulent flow has been observed in the aneurysm space after systole (Valen-Sendstad et al. 2011). Accordingly, the k–ω shear stress transport turbulence model (Menter et al. 1994) was adopted to realize the turbulence characteristics in the simulated intracranial circulation environment.

### 2.3 Simulations of an SMPf-Based Device Implementation

**Figure 2:**
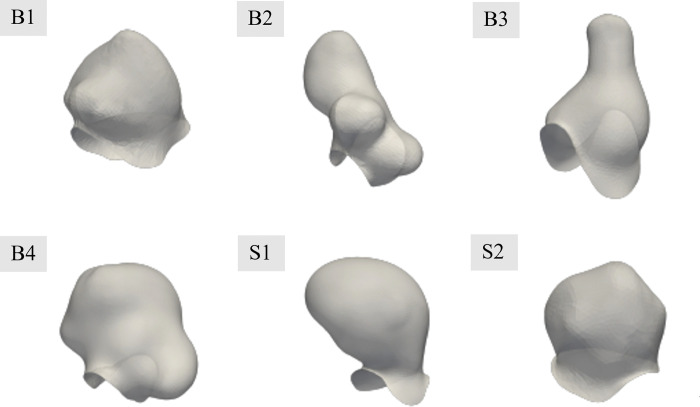
SMPf device geometry for each of the six patient-specific ICA cases examined. Devices are shown with slight transparency to represent the full neck region (views are shown at size for clarity but are not a consistent scale).

Treatment efficacy was evaluated by virtually implanting SMPf devices into each patient-specific aneurysm geometry. First, the geometry of each SMPf device was generated by manually excising the aneurysm sac from the surrounding vasculature (Fig. 2). The devices were then idealized and represented the maximum possible foam volume for the specified geometries. Manufacturing considerations or defects/discrepancies were not taken into account.

Because in this study we focused on providing general design suggestions rather than seeking to evaluate a patient-specific case in a clinical setting, we chose a continuum porous media approach to model the SMP foam. The porous medium specifications considered in this study followed the previous work by Muschenborn et al. (2013) with a porosity Φ=98%, permeability *k* = 3.25 × 10^−9^ ~ 1.24 × 10^−8^ m^2^, and form factor *C* = 2720 − 7260 m^−1^. These parameter ranges were verified by evaluating the pressure gradient of the flow through the porous media model at different flow velocities and comparing them with the original study (Muschenborn et al. 2013). For the results presented in this work, the lowest permeability model was selected because it demonstrated the greatest performance in reducing intra-aneurysmal flow.

### 2.4 Simulations of Heat Transfer Process During Initial Shape Recovery that Triggers Heating

**Figure 3:**
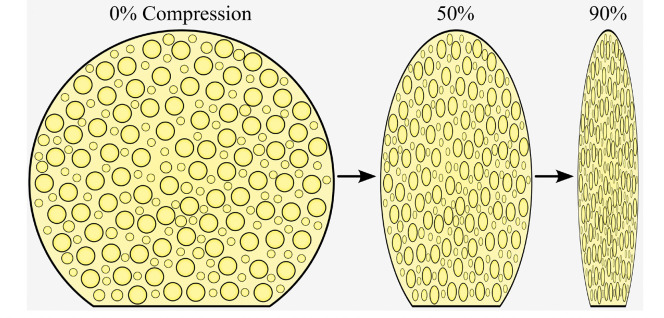
Schematic of the compression of a representative SMPf device in its original state at 50% and 90% radial compressive strain.

To quantify the thermal effects of device deployment and the heating associated with the initiation of shape recovery, an idealized, rigid-walled solid cylinder with a diameter of 1 mm was used. The use of a compressed solid model was justified because the pores of the closed cell SMPf were almost completely closed upon compression (Fig. 3). The length of the cylinder models varied between ICA cases, but in all cases ranged from the aneurysm neck to a 1 mm offset from the vessel walls perpendicular to the neck, centered in the intra-aneurysmal space.

The potential thermally induced tissue damage can be described by the cumulative equivalent minutes at 43°C (CEM_43_), defined by

1 $$CEM_{43}(t)=\int_{t_{0}}^{t_{f}}R^{\left(43-T(t)\right)}dt$$

Where $t_{0}$ is the initial time, $t_{f}$ is the end time of exposure in minutes, R is a constant of 0.5 for $T\;\left(t\right)\geq \;43{}^{\circ}C$, 0.25 for 39°C≤T(t)<43°C, and 0.0 for T(t)<39°C (Sapareto et al. 1984). According to van Rhoon et al. (2013), irreversible damage to brain tissues occurs with as little as CEM_43_=10. Assuming a constant exposure temperature, 40 °C and 45 °C stimuli would result in tissue damage after > 600 min and 2.5 min, respectively. Therefore, the SMPf device in this study was considered to have a glass transition temperature *T*g of 39 °C (Pineda-Castillo et al. 2021). Pineda-Castillo et al. (2021) have shown that the SMPf device can heat from well below body temperature to its transition temperature in less than a minute while completely occluding an *in vitro* aneurysm sac in the same amount of time. Thus, a fixed temperature of 40 °C was used in the heating portion of this numerical study, just above the *T*g of the SMPf material. A zero-heat flux (perfectly insulated) boundary condition was imposed on the vessel to model the worst-case temperature rise (Ortega et al. 2007). The flow through the inlet was set to body temperature (37 °C), with the blood being assigned a specific heat capacity of $c_{p}$ = 3600 J/kg· °C and a thermal conductivity of $k_{t}$ = 0.52 W/m· °C (Hasgall et al. 2018). The inlet flow for this study was modeled at the diastolic VFR of 2.98 mL/s and 1.77 mL/s for the internal carotid artery and basilar artery, respectively (Gwilliam et al. 2009) to minimize convection from the region and to simulate the highest possible temperature rise on the walls. The simulations were considered steady-state and included the Reynolds-averaged Navier-Stokes equations, although the slow flow rates significantly reduced the instabilities observed in the previous systolic flow simulations.

### 2.5 Simulations of Heat Transfer During Device Expansion

**Figure 4:**
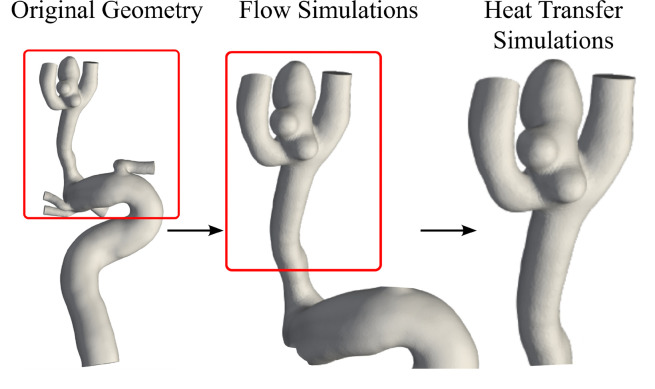
Geometry shortening for Case B2 in the original, flow simulation and heat transfer simulation states.

Next, transient heat transfer simulations were performed on a representative patient geometry, Case B2, which demonstrated the highest level of heat transfer during the initial heating stage of the deployment. To limit the computational expense of the additional solution steps, the domain upstream of the ICA was shortened to approximately three diameters of the parent artery (Fig. 4) and remeshed with a cell size of 0.20 mm to generate approximately 41,000 cells.

To provide a more realistic flow condition than the previous diastolic study, outflow VFRs were calculated from the mean ICA velocity. All other boundary conditions were retained. Although the simulation was transient, the flow was still modeled as steady-state rather than pulsatile, using a time step of 1 ms and a PIMPLE algorithm, an iterative solver which is a combination of PISO (Pressure Implicit with Splitting of Operator) and SIMPLE (Semi-Implicit Method for Pressure-Linked Equations). For computational efficiency reasons, the total time steps were limited to 1,000. The geometry was constructed as a partially expanded foam with an offset of 0.5 mm from the vessel wall. As in the flow simulations, the model was defined as a porous medium with a permeability of 5.7 × 10^−9^ m^2^ and form factor of 7260 m^−1^. The entire zone of the porous medium was assigned a temperature of 40 °C and 45 °C for the two respective shape recovery-induced heating processes to provide an optimal and worst-case heating scenario. The surrounding fluid volume was modeled similarly to that described in Section 2.4.

### 2.6 Simulation Verification

A mesh independence study was performed on the aneurysm B1 geometry (Fig. 1), with simulations covering a range of 0.05–0.16 mm cell sizes ( 6.87 × 10^5^ − 1.25 × 10^7^ cells). The number of cells was approximately doubled for each successive cell size, without the finest mesh containing 1.5 times as many cells as the second finest mesh. The maximum and average flow velocities for 1,000 points evenly distributed throughout the aneurysm domain were calculated in ParaView (Ahrens et al. 2005). To reduce the influence of insignificant errors/discrepancies in the simulation, the slowest 1% of the sampled points, corresponding to a velocity of 0.03 m/s, were excluded from the analyses.

To further verify the simulation parameters and results, the mean internal carotid artery flow rate of 5.11 mL/s (Gwilliam et al. 2009) was scaled and applied to a 0.08 mm mesh. This enabled a direct comparison of WSS values between this study and the time-averaged WSS results of the six most experienced teams at the 2015 International Aneurysm CFD Challenge (Valen-Sendstad et al. 2018). However, despite their experience, there was no clear consensus regarding the WSS magnitude numerically determined between the six teams: i.e., four teams achieved comparable values and the other two teams reported drastically different values. While a direct quantitative and numerical comparison may not have a high degree of validity in this case, qualitative contrasts can be drawn based on the WSS distributions presented by the teams as they are holistically more similar.

### 2.7 Simulation Metrics and Data Analysis

For the flow velocity studies, the WSS distribution was beneficial to qualitatively verify the validity of the flow within the model. Intra-aneurysmal velocity is also an important metric to evaluate since reducing flow is a necessity for rebuilding and recovering arterial tissue. Intra-aneurysmal velocity was evaluated for maximum and mean quantities for the interior of the aneurysm space. For the heat transfer studies, the temperature for the intra-aneurysmal space and surrounding vessels was assessed, and visualized using isosurfaces and contours. For all studies presented, ParaView (Ahrens et al. 2005) was utilized to extract the above metrics from the simulated data and generate visualizations. The Python library Matplotlib (Hunter 2007) was used to generate the CEM_43_ plots.

## 3. Results

### 3.1 Study on Mesh Size

**Figure 5:**
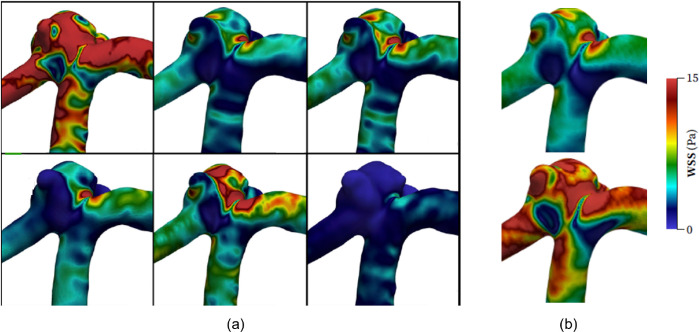
Comparison of WSS values to representative results from the 2015 International Aneurysm CFD Challenge (Valen-Sendstad et al. 2018): (a) WSS distributions for the six highly experienced teams (units are in Pa, and layout rearranged); (b) WSS distribution calculated for mean flow rate (*top*) and systolic flow rate (*bottom*).

The mesh size was determined to be *s* = 0.08 mm as this was the maximum cell size with less than 5% mean absolute error (MAE) compared to the control (*s* = 0.05 mm), resulting in a cell density of approximately 1,700–1,800 cells/mm^3^, which is consistent with the cell densities presented in the literature (Marzo et al. 2009; Hodis et al. 2012; Aranda et al. 2018). The mesh convergence study results are summarized in Table 2. Further qualitative comparisons of the WSS contours with those of the 2015 International Aneurysm CFD Challenge (Valen-Sendstad et al. 2018) showed good agreement in the WSS distributions along the aneurysm surface (Fig. 5).

### 3.2 Simulations of Untreated and Treated ICAs

As discussed in Section 2.3, porous media models were used to define the permeability of the SMPf device. For the results presented here, the lowest permeability of the foam was adopted, defined by a permeability of 5.7 × 10^−9^ m^2^ and a form factor of 7260 m^−1^. In each case, treatment of the aneurysm showed significant decrease in blood flow into the aneurysm cavity. Overall, in each case, the maximum flow velocity was reduced by > 40% and the average flow velocity was reduced by up to 95%, as shown in Table 3. The metric used to quantify this reduction was the velocity reduction ratio VRR, i.e.

2 $$VRR=\frac{u_{pre}-u_{post}}{u_{pre}},$$

where u is the predicted fluid velocity and the subscripts, pre and post, denote the respective velocities before and after device implantation, respectively.

**Figure 6:**
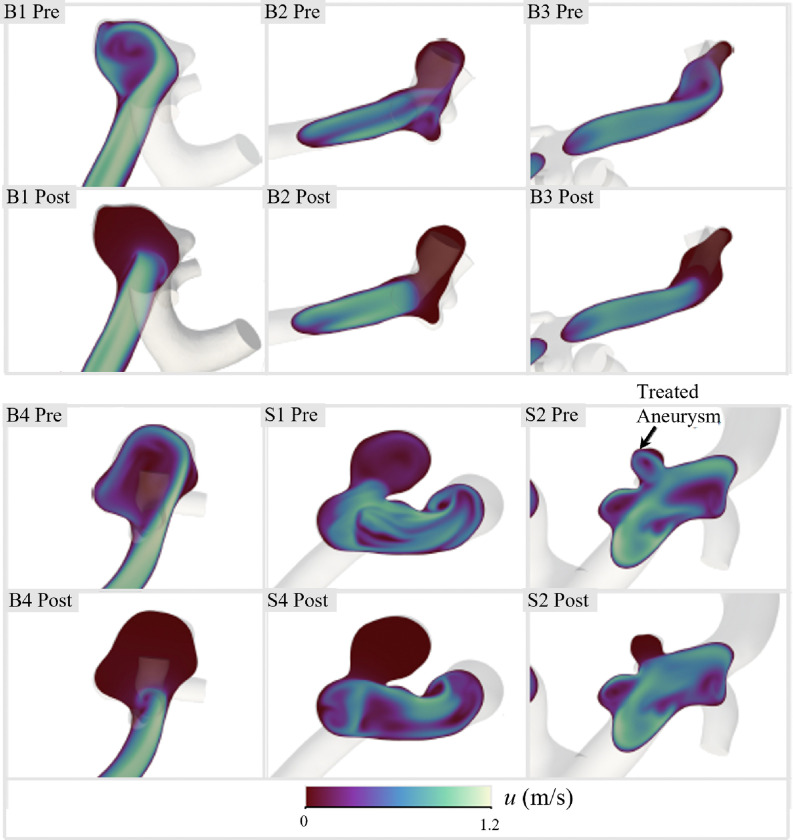
Comparison of intra-aneurysmal velocities before and after treatment in the central plane of each of the 6 ICAs studied.

Furthermore, this residual flow reduction can be visualized using the velocity contours of the central aneurysmal plane and inlet vessel for both the untreated and treated cases (Fig. 6). More profound differences were found in the bifurcation cases (B1, B2, B3, and B4), where high-velocity inflow jets were observed prior to treatment. In addition to reducing flow within the intra-aneurysmal space, device implantation appeared to have stabilized the downstream flow, as indicated by the smoother velocity contours in these areas. While the flow reduction results are promising for high efficacy of a patient-specific SMPf-based device, the deployment of these devices introduces several biocompatibility concerns, with thermal damage to surrounding arterial tissue being the greatest concern and requiring further investigation.

### 3.3 Heat Transfer Simulations for Initial Heating of the SMPf Device – Triggering Shape Recovery

**Figure 7:**
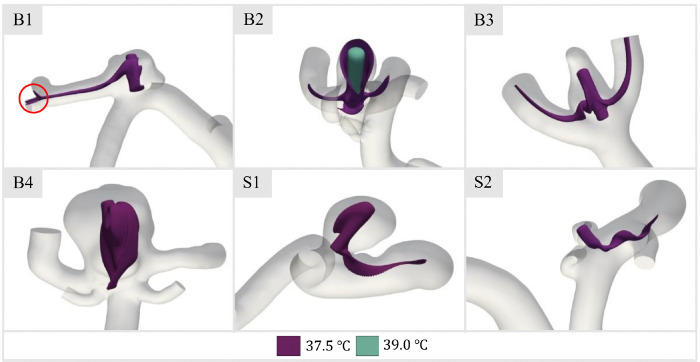
Temperature isosurface of 37.5 °C for steady-state heating in patient-specific aneurysm geometries. Note the inclusion of a second isosurface (*T*= 39 °C) for Case B2 and the indicated downstream isosurface contact for Case B1.

As described previously, arterial tissue damage occurs near CEM_43_ of 10 minutes (van Rhoon et al. 2013). Therefore, the thermal effects of the device deployment process on the aneurysm and artery wall tissue must be carefully investigated to identify possible thermal damage with respect to the appropriate activation temperature for shape recovery. The first case considered was the initial thermal activation heat transfer, as at this point the convection patterns from the SMPf device to the surrounding fluid became visible. Temperature isosurfaces were plotted to observe heat convection and the distribution of the initial activation heat to the surrounding fluid (Fig. 7). The selected isosurface was chosen at 37.5 °C just 0.5 °C above body temperature, to maximize interpretable data of the underlying heat transfer process and impact.

In general, the 37.5 °C isosurfaces (Fig. 7) did not contact any walls within the aneurysm space or downstream vasculature, which was expected given the minimal temperature gradient generated during device activation at 40 °C. However, there are two exceptions. In case B1, a thin stream of heated fluid impinges on the wall at a downstream bifurcation, although heat transfer thereby is negligible. In addition, given the low fluid velocities compared to the other cases (Fig. 6), it was not surprising that Case B2 was somewhat more concerning as it was found that convection within the dome was significantly hindered. This allows fluid at a higher temperature level to come into contact with the vessel wall at the apex of the treated aneurysm. This resulted in an open isosurface at 37.5 °C, indicating local wall temperatures above body temperature. Nevertheless, with the 39 °C isosurface superimposed, the temperatures on the vessel wall were still lower than those of the device ( 40 °C). Because Case B2 showed the greatest concern for exposing vessel walls to potentially degenerative temperatures, this case was utilized to further explore the impact of heat transfer during the subsequent expansion phase of the device.

### 3.4 Transient Heat Transfer During SMPf Device Expansion and Assessment of Tissue Damage

**Figure 8:**
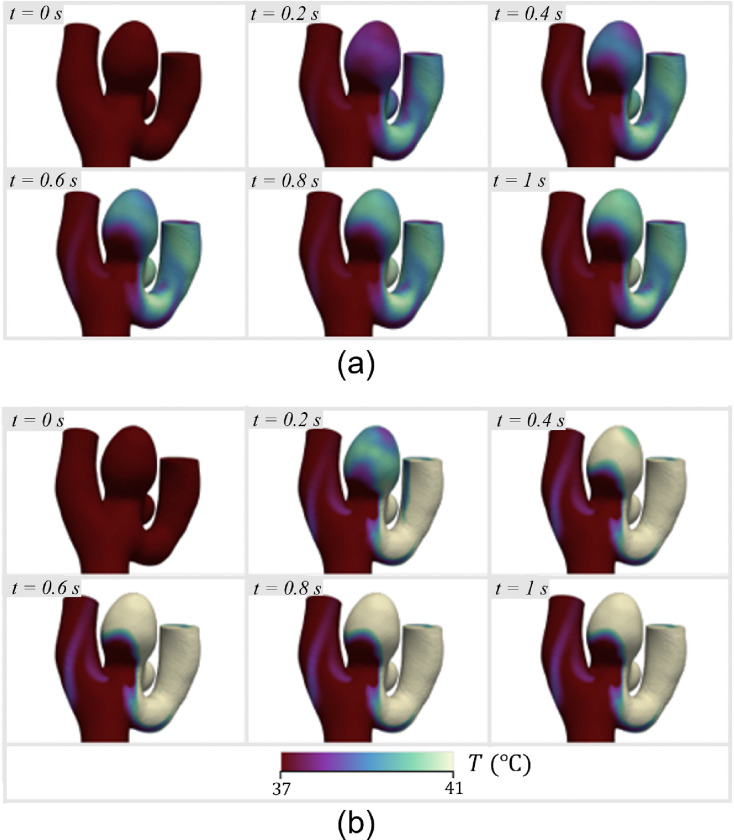
Temperature at the vessel walls over 1 s of the expansion and heating process, starting with a 40% expanded foam: (a) 40 °C stimulus; (b) 45 °C stimulus.

The maximum wall temperature was observed to increase to 40 °C within 1 s at both stimulus temperatures, which is due to the relatively slow flow within the aneurysm dome and the small distance between the SMP foam and the tissue wall (Fig. 8). It was found that the blood flow pattern is such that only certain regions of the aneurysm sac reach elevated temperatures, while almost all of the heated blood from those areas drains into only one of the bifurcation vessels, significantly increasing its wall temperature.

**Figure 9:**
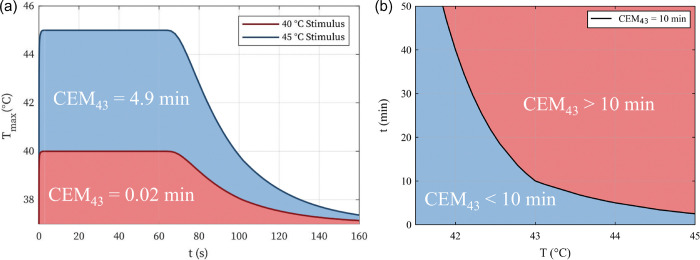
Analysis of potential thermal damage caused by the shape recovery heating stimulus: (a) time history of the maximum wall temperature of the simulated stimulus for the triggering of the SMPf shape recovery and the resulting cooling; (b) representative temperature *T* and time *t* combinations related to CEM_43_ = 10 min (blue = no thermal damage; red = thermal damage).

The time histories for the heating and cooling process of the SMPf device shape recovery can be combined to calculate the CEM_43_ dosage for Case B2. Assuming a total heating time of 1 minute (Pineda-Castillo et al. 2021), the maximum vessel wall temperature curves were generated throughout the entire shape recovery process (Fig. 9(a)). Since the blood flow temperature for the 40 °C stimulus was found to never exceed 40 °C, the CEM_43_ measurement is almost negligible for the entire heating process (i.e., 0.02 min). In contrast, the 45 °C stimulus resulted in a worst-case CEM_43_ of 4.9 min. Even in this (over-heating) worst-case scenario, there would be no thermal damage to the surrounding arteries/aneurysm wall (van Rhoon et al. 2013). The general relationship between time and temperature for CEM43 is shown in Fig. 9(b).

## 4. Discussion

In this study, we examined the viability of a patient-specific SMPf device for the treatment of ICAs. We explored the hemodynamical effects of device implantation on intra-aneurysmal flow as well as the implications of a thermal heating mechanism within the aneurysm cavity, taking into account both the initial heating stages and transient heating during device expansion. Below we will discuss the potential advantages and disadvantages regarding the efficacy of the proposed SMPf patient-specific embolic device and the validity of the numerical studies presented here.

### 4.1 Flow Simulations

In this work, we presented baseline and treatment flow simulations for six different patient-specific geometries, with the SMPf device modeled as a porous medium. In the untreated cases, we found an average maximum velocity in the aneurysm space of 0.94 ± 0.18 m/s and an overall average velocity of 0.32 ± 0.12 m/s across all patient geometries. After treatment, we found an average maximum velocity of 0.40 ± 0.07 m/s and an overall average velocity of 0.031 ± 0.017 m/s resulting in an approximate reduction of 60% and 90% in the maximum and average flow velocities, respectively. While the low permeability foam used in our numerical simulations showed promising results, in real practical applications higher permeability may be required to meet the required compression constraints as the device compresses radially and is inserted into an endovascular micro-catheter with an inner diameter of < 1 mm for use in clinical setting.

In our internal pilot studies, not presented here, we found that higher permeability foams do not perform as well as the idealized low permeability foam presented in this manuscript. A study by Ouared et al. (2016) found that with vascular stents, even a 30% reduction in intra-aneurysmal flow can promote thrombosis in the aneurysm sac, which can ultimately lead to effective aneurysm occlusion. Therefore, even with higher permeability foam, there is still evidence for the viability of the SMPf device presented. In addition, Ortega et al. (2013) evaluated an SMPf device with higher permeability (*k* ≈ 2 × 10^−8^ m^2^) and found a flow reduction in the intra-aneurysmal region furthest from the aneurysm neck up to 95%. Their results are consistent with our results presented in this work.

While endovascular coil embolization remains one of the most prominent methods for the endovascular (minimally invasive) treatment of ICAs, the SMPf device offers potential solutions to some of the more common limitations of GDC coiling therapeutic. Most importantly, the patient-specific SMPf aims to maximally reduce the residual volume of the aneurysm, which is a cause of recurrence in patients treated with coils and requires repeated surgery (Griessenauer et al. 2016). Another shortcoming of coil embolization that the proposed SMPf device aims to improve is post-treatment compaction. Because the soft platinum wires used in coiling are delicate and flexibly deformable, clinical evidence has shown that blood inflow into the aneurysm sac can gradually compact the packed coils, further leading to residual volume near the aneurysm neck and causing recurrence (Yu et al. 2019). On the other hand, the SMPf device would have a higher degree of structural integrity due to its highly interconnected cellular structure.

### 4.2 Device Heating and Deployment

Heat transfer from SMPf device deployment and initiation of shape recovery is highly dependent on the specific aneurysm geometry and flow speed (Fig. 7). For example, transient analysis of Case B2, which showed the highest level of heat transfer from the device to the aneurysmal and arterial walls, indicated that the wall temperature would approach that of the expanding SMPf within seconds. While this was most clearly observed in Case B2, it is likely that this condition applies to all study cases during further stages of device expansion as fluid is forced from the intra-aneurysmal space and convection capacity is reduced. However, the potential thermal damage to tissue from this heating process depends heavily on the temperature required for the device to expand (e.g., a thermal activation threshold very close to body temperature would still not cause damage to surrounding tissue regardless of the duration of the activation/expansion process). Furthermore, the heterogeneity of the arterial tissue composing the aneurysm sac, both in terms of microstructural composition and tissue thickness (Laurence et al., 2021), may result in a non-uniform response to the heating stimulus. This can potentially lead to local thermal damage below the previously mentioned stimulus thresholds.

Other possible solutions to the heating problem include reducing the time required for full expansion, which is also related to the material properties of the SMPf, as the discrepancy between the temperature provided to the device and the *T*g of the device regulates the rate at which expansion occurs (Kunkel et al. 2018; Shahi et al. 2022). This positive correlation between shape recovery rate and stimulus temperature is related to the increased kinetic energy within the polymer system at higher temperatures, which allows the polymer chains to move more freely and reestablish their initial geometric state more quickly (Davidson et al. 2015). Furthermore, although the boundary conditions presented in this study provided the stimulus temperature (40 °C and 45 °C) on the exterior of the SMPf model, in practice with a central heating element, the low thermal conductivity of a SMPf (Soloveva et al. 2022) would likely have a strong thermal gradient that reduces heat transfer to the blood and surrounding tissues during all stages of deployment.

### 4.3 Study Limitations and Future Work

Although the results presented in this study are promising, there are notable limitations that should also be discussed. First, no experimental validation was performed to verify the accuracy of the presented results. Second, the porous models presented in the study were all isotropic in nature, ignoring the potential effects of anisotropic foam, which are more common in practice due to the foaming process (Ortega et al. 2013). Furthermore, the steady-state assumption neglected potential benefits of the foam-based device, e.g., previous research studies have shown that SMPf can filter out high-frequency velocity fluctuations in the aneurysm space (Ortega et al. 2013).

In short, the SMPf device promises to reduce intra-aneurysmal flow velocities and offers potential solutions to the limitations encountered with traditional coil-based methods. Now we will discuss some possible drawbacks arising from the thermal activation mechanism for SMPf deployment. The most urgent extension of this work is the construction of an *in vitro* flow loop with an integrated particle image velocimetry system for experimental validation of the studies reported herein. Moreover, extensions of computational simulations with pulsating, time-dependent conditions can provide insights into flow behavior caused by inertial effects not foreseen in this work. An analysis of the deployment of the device would be helpful in determining the viability of the underlying concepts of a SMPf device (e.g., examining the stresses that the expanding foam exerts on the vessel wall in the event of device misalignment, similar to the work of Hwang et al. 2012). Further analysis of the thermal properties of the specific SMPf material and inclusion in the aforementioned heat transfer simulations would provide a more realistic description of the plausibly harmful heat generation of the device during expansion in the intra-aneurysmal space.

Another possible extension is the inclusion of a fluid-structure interaction (FSI) approach to model blood flow-induced deformations in the aneurysm and foam. With such an approach, the surfaces are not rigid and can respond to fluid motion. Accordingly, FSI modeling is more computationally expensive and requires a higher degree of boundary conditions and model data since both the fluid and solid domains are modeled. If properly performed, FSI modeling would offer a more accurate physiological representation of the blood flow patterns in the ICA and the effects of the proposed SMPf device on it.

### 4.4 Conclusion

In this work, we presented results on the hemodynamic effects and possible heat transfer impairments of a SMPf device for the treatment of ICAs. We have demonstrated a significant flow reduction in the intra-aneurysmal space, effects of initial shape recovery on various aneurysm geometries, and, in the worst case, transient heat transfer to the surrounding tissue. Although these results were promising and prospective solutions have been developed to address concerns about device heat transfer during shape recovery induction, future work is still required to address the full complexity of treating ICAs and develop a highly effective treatment device. However, this work adds to the growing body of research assessing the effectiveness of SMPf devices for the endovascular treatment of ICAs, thereby providing additional evidence for further investigation and the design and development of personalized SMPf-based endovascular devices.

## Figures and Tables

**Table 1:** Characteristics of the six patient-specific aneurysms studied (B1-B4 and S1, S2).

	Location	*I*_max_ (mm)	AR	SR	*V*(mm^3^)	*A*_ostium_ (mm^2^)
B1	MCA bifurc.	7.7	1.0	1.8	76	28.2
B2	MCA bifurc.	7.8	1.5	2.1	50	12.4
B3	MCA bifurc.	5.6	1.6	1.9	41	21.0
B4	Basilar apex	11.1	1.3	2.5	313	36.1
S1	Sup. hypophyseal	9.0	2.3	2.0	167	24.4
S2	Ant. choroidal	2.9	0.8	0.4	10	6.6

MCA birfurc. = middle cerebral artery bifurcation; Sup. hypophyseal = superior hypophyseal artery; Ant. choroidal = anterior choroidal artery; *I*_max_ = facet diameter; AR = aspect ratio (sac depth: neck width); SR = size ratio (sac depth: parent artery diameter); *V* = volume of aneurysm sac; *A*_ostium_ = interface area between aneurysm sac and main vessel.

**Table 2:** Detailed results of the mesh independence study. (Velocity values are calculated over the entire aneurysm domain.)

*s* (mm)	Cells (×10^3^)	IA cells (×10^3^)	*u*_max_ (m/s)	*u*_avg_ (m/s)	MAE (%)
0.05	12450	938	1.106	0.494	–
0.06	7780	612	1.103	0.494	1.5
0.08	4160	329	1.097	0.494	4.4
0.10	2230	194	1.086	0.494	9.0
0.13	1150	106	1.061	0.490	15.9
0.16	687	67	1.054	0.490	18.3

*s* = mesh size; IA = intra-aneurysmal; *u*_max_ = maximum velocity; *u*_avg_ = average velocity; MAE = mean absolute error.

**Table 3:** Simulation results of intracranial flow velocity before and after SMPf implantation. All velocity values are given in m/s.

	*u*_max,pre_	*u*_max,post_	*VRR*_max_	*u*_avg,pre_	*u*_avg,post_	*VRR*_avg_
B1	1.107	0.456	58.8%	0.494	0.054	89.0%
B2	0.746	0.387	48.2%	0.172	0.017	90.0%
B3	0.775	0.333	57.1%	0.328	0.033	90.1%
B4	1.180	0.488	58.7%	0.333	0.022	93.3%
S1	0.997	0.428	57.1%	0.219	0.011	95.0%
S2	0.825	0.329	60.1%	0.403	0.048	88.0%

*u* = predicted fluid velocity; pre = before device implantation; post = after device implantation; *VRR* = velocity reduction ratio;

## Data Availability

Data is provided within the manuscript or supplementary information files.
